# Generic Drug Usage in Dentistry across Japan: Analysis Using a Japanese National Database

**DOI:** 10.3390/ijerph182111329

**Published:** 2021-10-28

**Authors:** Sachie Ono, Akira Komatsuzaki, Asami Iguchi, Hitomi Kikuchi, Shiho Motoi, Mio Susuga

**Affiliations:** 1 Department of Preventive and Community Dentistry, School of Life Dentistry at Niigata, The Nippon Dental University, 1-8 Hamaura-cho, Chuo-ku, Niigata 951-8580, Japan; sachie@ngt.ndu.ac.jp; 2 Department of Dental Hygiene, College at Niigata, The Nippon Dental University, 1-8 Hamaura cho, Chuo-ku, Niigata 951-8580, Japan; hitomi@ngt.ndu.ac.jp (H.K.); hsjc@ngt.ndu.ac.jp (S.M.); mio@ngt.ndu.ac.jp (M.S.); 3 Department of Dental Anesthesiology, School of Life Dentistry at Niigata, The Nippon Dental University, Chuo-ku, Niigata 951-8580, Japan; asami@ngt.ndu.ac.jp

**Keywords:** generic drugs, healthcare costs, dental care, health insurance, regional disparities

## Abstract

The aim of this study was to identify regional disparities in generic drug usage and to examine related factors. The database used for the analysis was the 2018 national health insurance claims data published on the Japanese Ministry of Health, Labour, and Welfare. The drugs that were targeted were a combination of brand-name and generic tetracycline ointments for periodontal treatment and lidocaine injection solution used for dental anesthesia. The usage of generic drugs was calculated and compared by prefecture based on the number of health insurance claims. The comparison of related factors was conducted using data from other national statistical survey. The results showed that the mean generic drug usage of tetracycline for periodontal treatment in all prefectures was 71.2 ± 8.1%, ranging from 45.8% to 85.3%. The mean generic lidocaine used for dental anesthesia was 47.6 ± 10.0%, ranging from 30.5% to 66.2%. The rank correlation coefficient between the two was 0.359 (*p <* 0.05), and the tendency of using both generic drugs was low in major metropolitan areas. Generic drug usage in Japan is low; thus, in order to reduce healthcare costs, generic drugs need to be actively used in dentistry.

## 1. Introduction

Generic drugs play an important role in sustaining the modern healthcare system, because escalating healthcare and drug costs are becoming a growing problem worldwide [1]. Healthcare costs in Japan have been escalating since the 1960s, presumably due to the establishment of the universal health insurance system and the subsequent increase in the number of patients receiving medical care, as well as the rapid aging of the population [2].

Concerned about the financial impact of rising healthcare costs, the Japanese government started introducing generic drugs from an early stage; however, the share of generic drugs in Japan did not increase as much as in Western countries. In 2011, the share was only 22.8%, and Japan was advised by the Organization for Economic Cooperation and Development (OECD) to increase the share of generic drugs to 30% or more [3].

In response to this situation, to facilitate medical institutions making the transition to generic drugs, health insurance schemes introduced the “addition of insurance points”. Prescription formats were also changed to make it easier for patients to request a change to generic products. The usage of generic drugs has increased to about 70% in recent years as a result of the Ministry of Health, Labour and Welfare’s (MHLW) policies to expand the use of generic drugs, which is expected to remain an effective tool for reducing healthcare costs in the future [4].

On the other hand, since prescription drugs are limited in dentistry, we believe that it is important to ensure management stability of dental clinics by increasing the use of therapeutic generic drugs. The decline in the number of edentulous elderly patients should be welcomed; however, the number of dental treatments is expected to increase as the remaining teeth are at relatively high risk for disease. The context of medical costs in dentistry is not so simple. Since the interests of pharmaceutical companies, insurance pharmacies, and health insurance-covered medical institutions related to generic drugs are intricately intertwined, the government, which is responsible for providing the institutional framework, finds it difficult to take forceful measures [5]. To sustain universal health coverage in the future, it is important to change the mindset of medical professionals.

Globally, the analysis of generic drug usage has been increasing since the 1980s, which is said to have been triggered by the implementation of the Hatch-Waxman Act (Drug Price Competition and Patent Term Restoration Act) in the United States [6], and the generic market has grown rapidly since 1990. An overview of the economic benefits has been reported by Caves et al. [7] and Reiffen et al. [8]; however, only the study by Hellerstein [9] mentioned regional disparities. The only reported study of generic drugs in dentistry was reported by Sharma [10], which did not mention regional disparities. The OECD 2017 report also pointed out that usage should be improved [11].

Thus, to provide basic data for future measures, this study used data from a government-published health insurance database and examined the regional disparities in generic drug usage in the field of dentistry and its related factors.

## 2. Materials and Methods

### 2.1. Study Design and Resource Database

A cross-sectional study design was adopted, and data from a single fiscal year obtained from a government-published database were used. In Japan, the MHLW has taken the initiative to publish health insurance data in stages and make them available for researchers. Data (number of health insurance claims by item related to dentistry from April 2018 to March 2019) from the most recently published 5th National Database of Health Insurance Claims and Specific Health Checkups of Japan (NDB) were used as the data source.

### 2.2. Study Methods

**Figure 1:**
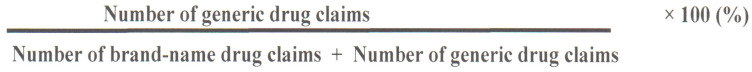
Formula for calculating the percentage of generic drug usage. The data used were the number of claims for the period between April 2018 and March 20219. For the correlation analysis, both of the values (upper limit 200%) were used for dental use (in-hospital).

The number of claims for generic and brand-name tetracycline ointments for periodontal treatment (in total, 3,310,935 cases) and lidocaine injection solution used for dental anesthesia (in total, 3,315,728 cases) (Table 1) was obtained from the 5th NDB Open Data (2018 national health insurance claims data) published on the MHLW website, and the percentage of generic drug usage was calculated based on the number of health insurance claims (Figure 1). To compare therapeutic and prescription drugs in dentistry, 60 mg non-steroidal-anti-inflammatory-drugs (NSAIDs) tablets and six generic drugs were compared (in total, 19,990,343 cases, approved for tooth aches).

To analyze factors related to the usage of generic drugs, Spearman’s rank correlation coefficient matrix was calculated to examine the association with items such as national statistics findings (e.g., Survey of Physicians, Dentists and Pharmacists) by prefecture (e.g., number of dentists per 100,000 population) and total amount of dental insurance claims. A scatter plot was also created to compare the distribution among the items.

For the statistical analysis, Microsoft Excel (Microsoft Japan, Tokyo, Japan) was used to calculate generic drug usage and the analysis of the contingency tables, and BellCurve for Excel (Social Survey Research Information Co., Ltd., Tokyo, Japan) was used for the correlation analysis.

### 2.3. Study Ethics

The present study was conducted after obtaining approval from the Research Ethics Committee of Nippon Dental University (approval No.ECNG-R-5). A national database was used as the data source. Although the government obtains informed consent from subjects and the methods used are not disclosed, the methods proposed in the Japanese national statistics were followed, and the protocol complied with the Declaration of Helsinki. Since this study was based on publicly available population data, personal information was not included in the analysis.

## 3. Results

**Figure 2:**
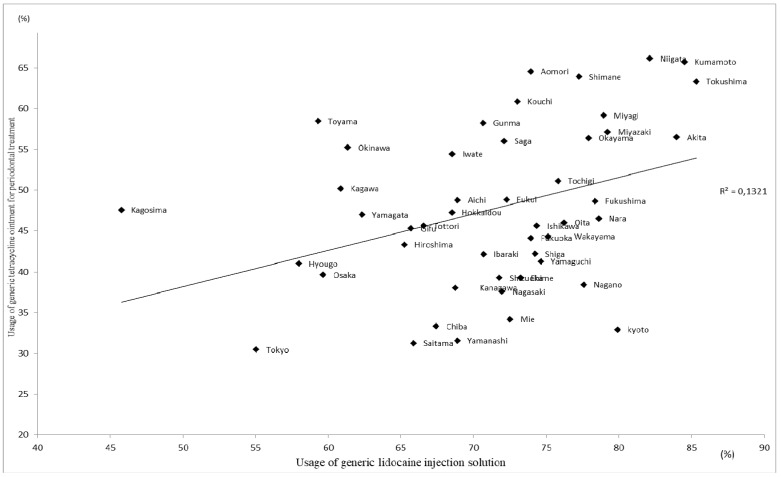
Comparison of usage of generic tetracycline ointment and lidocaine injection solution.

**Figure 3:**
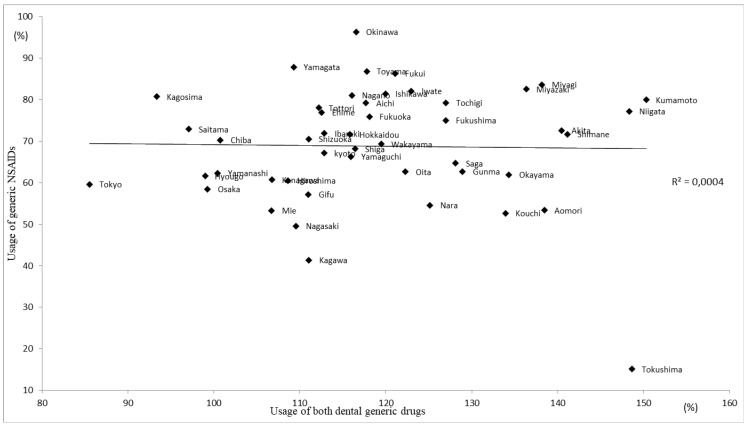
Comparison of usage of NSAIDs and both in-hospital generic drugs.

The mean percentage ± standard deviation (SD) usage of generic tetracycline ointment for periodontal treatment by prefecture was 71.2 ± 8.1%, with a range between 45.8% and 85.3%. The mean value ± SD for generic lidocaine injection solution used for dental anesthesia was 47.6 ± 10.0%, with a range between 30.5% and 66.2%. The rank correlation coefficient between the two was 0.359 (*p <* 0.05), and it tended to be low in metropolitan areas such as Tokyo, Hyogo, and Osaka (Figure 2). Both were high in areas such as Kumamoto, Tokushima, and Niigata prefectures. In comparison, the mean percentage ± SD usage of outpatient (non-hospital) generic NSAIDs tablets was 68.8 ± 14.1%, with a range between 15.1% and 96.3, and there was no correlation between the two outpatient (in-hospital) drugs (Figure 3). Each relationship figure shows the coefficients analyzed by simple regression line analysis from each prefecture item.

Items correlated with the percentage usage of generic drugs from the results of the rank correlation matrix (Table 2) were items such as consultation days (−0.433; *p* < 0.01) and total dental care cost (−0.444; *p <* 0.01), and no correlation was found with items such as healthcare cost per day, number of dentists per 100,000 population, and usage of generic NSAIDs. This section may be divided by subheadings. It should provide a concise and precise description of the experimental results, their interpretation, as well as the experimental conclusions that can be drawn.

The negative correlation observed between generic drug usage and the relevant factors related to healthcare costs (consultation days and total dental care costs) and the characteristics of the scatter plot showed that dental care costs tended to be higher in areas with low generic drug usage.

## 4. Discussion

The analysis of the present study showed that, in the field of dentistry in Japan, there are regional disparities when it comes to the use of generic drugs.

It is thought that increases in drug costs are greatly impacted by the amount of drug administered or prescribed and the rise in prices; however, there are many uncertainties as to whether this impact extends to the regional level. Health insurance schemes, including drug usage, in many countries are often run at the national level, and policies recommending the use of generic drugs are often proposed by the government [12].

However, a noteworthy aspect in Japan is that the health insurers in a position most likely to promote the reduction of healthcare costs are found at the regional level. Moreover, organizations such as clinics and pharmacies, which have decision-making authority related to the usage of generic drugs, are organized at the prefectural level, and many of the audits related to medical fees covered by health insurance are also conducted at the regional level.

These aspects, both in the field of medicine and dentistry, are among the characteristics of the Japanese health insurance system, and although it is a universal health insurance system, the presence of multiple health insurers has a complicating effect.

Iizuka et al. cited the effect of drug price differences and problems in the healthcare system as factors affecting generic drug usage in Japan [13]. In addition to the influences of incentives, Ohashi et al. cited factors such as the effects of regional disparities in income [14]. Although both studies were based on analyses of outpatient medical prescriptions, it is possible that dentistry has been affected in a similar manner.

Since symmetrical trends were particularly observed between urban and rural regions, further studies related to a region’s economic strength may be needed in the future. Increased copayment in public health insurance in regions with weak funding has already become a problem [15].

As for factors related to dental care providers, there are fewer available generic dental drugs compared to those for medical use, and even when they are available on the market, problems may exist on the supply side, and some generic drugs have more limited indications than brand-name drugs [16]. As for therapeutic drugs, the problem may be that patients do not immediately see the benefits of switching to generic drugs, since the system is designed so that profits are absorbed by the medical institutions.

However, since the accrued difference from the switchover contributes to the improvement of management of medical institutions, it can be said that the management efforts of dental clinics are reflected in the usage of generic drugs. According to the analysis of the present study, the Tokushima and Niigata prefectures, which tended to have high generic drug usage, had more dental clinics per population than other regions and were, therefore, in more competitive environments [17].

In dental health measures for the elderly with high healthcare costs, the focus of preventive measures is shifting from dental caries prevention to preventing periodontal disease. Periodontal disease has been associated with many diseases such as diabetes mellitus [18], and since it follows a chronic pathological course, polypharmacy needs to be addressed when it comes to drug usage. Although not all prescriptions of multiple drugs constitute polypharmacy, Kojima et al. reported that there are higher risks of adverse events in cases where six or more types of drugs are prescribed [19]. When we previously analyzed disease names for outpatient visits using Japanese national statistical data, we found that many patients with dental diseases were also visiting medical institutions [20,21], suggesting that there is a need to survey the status of administered drugs, including generic drug usage, from the patient’s side.

Furthermore, to promote generic drug usage in the future, Authorized Generics (AGs) also need to be promoted [22]. AG approval improves the image of the quality of generic drugs, and we think that usage may be affected if a user’s sense of security increases. To promote self-medication in Japan, OTC versions of conventional prescription drugs such as pain killers are being promoted [23]. However, from a dental practice perspective, we are concerned about the risk of losing the opportunity for early treatment. It is doubtful whether this generally leads to a reduction in healthcare costs.

The limitations of the present study were the cross-sectional design that used prefectural data from a single fiscal year and the scarcity of the number of drugs used to evaluate usage; thus, there may have been a difference from the overall trend in generic drug usage. Furthermore, since the analysis was limited to areas covered by health insurance, there may have been a larger disparity in major metropolitan cities where medical treatments not covered by insurance are more common. In order to resolve these issues, a large-scale survey of medication usage of healthcare costs is needed [24].

Promotion of generic drugs and the elimination of regional disparities are issues that should be addressed simultaneously to optimize healthcare costs, and we need to verify whether the implementation of a nationwide policy is needed. The findings of the present study showed that there is an especially large disparity between large metropolitan areas and rural regions, and the fact that there was a correlation with the environment of recipients suggested that we should focus on regional backgrounds.

## 5. Conclusions

Generic drug usage in the field of dentistry showed certain trends that varied by prefecture. Although an association with factors such as dental consultation trends in the region was suggested, the trends varied depending on the drug. In order to promote generic drugs in the field of dentistry, regional backgrounds need to also be examined.

## Figures and Tables

**Table 1:** Drug combinations used to calculate the percentage of generic drug usage.

Generic Category	Outpatient (in-Hospital) Drug for Dental Use	Drug Price (¥)	Number of Case
Brand-name	Tetracycline ointment [P1] 10 mg, 0.5 g	598.1	1,944,889
Generic Product	Tetracycline ointment [P2] 2% 10 mg, 0.5 g	399.5	1,370,839
Generic Category	Outpatient (in-hospital) drug for dental use: Dental local anesthetic	Drug Price (¥)	Number of case
Brand-name	Lidocaine Cartridge for Dental Use [L1] 1.8 mL	78.2	2,238,393
Generic Product	Lidocaine Inj. Cartridge [L2] 1.8 mL	58.0	1,072,542
Generic Category	Outpatient drug for dental use: Anti-inflammatory drug	Drug Price (¥)	Number of case
Brand-name	NSAIDs Tablets [N1] 60 mg	14.5	4,353,108
Generic Product	NSAIDs Tablets [N2-N6] 60 mg	7.8	15,637,235

**Table 2:** Correlation analysis between usage of each generic drug and indicators (Spearman’s rank correlation coefficients).

Spearman’s Rank Correlation Coefficient Matrix	Usage of Dental Generic Drugs	Consultation Days	Total Amount of Dental Claims	Dental Care Cost per Day	Number of Dentists per 100,000 Population	Percentage of Dentists Aged 60 Years and over	Number of Clinics per 100,000 Population
Usage of generic NSAIDs	0.188	−0.099	−0.129	−0.295 *	−0.313 *	0.105	−0.508 **
Number of clinics per 100,000 population	−0.300 *	0.461 **	0.469 **	0.131	0.801 **	−0.328 *	
Percentage of dentists aged 60 years and over	0.262	−0.596 **	−0.603 **	−0.106	−0.494 **		
Number of dentists per 100,000 population	−0.203	0.548 **	0.559 **	0.183			
Dental care cost per day	0.053	−0.071	−0.034				
Total amount of dental claims	−0.444 **	0.999 **					
Consultation days	−0.433 **						

(** *p <* 0.01, * *p <* 0.05).

## Data Availability

NDB open data: 2017 data: https://www.mhlw.go.jp/content/12400000/000711946.xlsx (accessed on 20 April 2021).
